# Occupational fatigue and its determinants among endoscopy nurses in China: a cross-sectional study with structural equation modeling

**DOI:** 10.3389/fpubh.2026.1759966

**Published:** 2026-02-04

**Authors:** Zhi Zeng, Yazhi He, Xiang Liao, Yu Song, Sumei Zhou

**Affiliations:** 1 Endoscopy Center, Deyang People’s Hospital, Deyang, Sichuan, China; 2 Department of Neurosurgery, Deyang People’s Hospital, Deyang, Sichuan, China

**Keywords:** coping style, endoscopy nurses, occupational fatigue, social support, structural equation modeling, work–family conflict

## Abstract

**Background:**

Occupational fatigue among nurses has become a critical global public health concern. Endoscopy nurses, in particular, face unique occupational demands that significantly impact their physical and psychological well-being, as well as the quality of patient care. This study aimed to assess the prevalence of occupational fatigue among endoscopy nurses in China and examine its associated factors. Specifically, the mediating role of coping styles in the relationship between work–family conflict and occupational fatigue was analyzed, and the influence of social support was explored.

**Methods:**

A multicenter cross-sectional study was conducted involving 332 endoscopy nurses from 26 tertiary hospitals across 14 provincial regions in China. Standardized instruments were used to measure occupational fatigue, work–family conflict, coping styles, and perceived social support. Descriptive statistics, univariate analyses, Pearson correlations, multiple linear regression, and structural equation modeling (SEM) were applied.

**Results:**

Endoscopy nurses reported a high level of occupational fatigue (22.075 ± 2.588). Age, gender, marital status, daily working hours, and weekly working days were significantly associated with fatigue levels (*p* < 0.05). Work–family conflict was positively correlated with occupational fatigue, whereas social support and positive coping were negatively correlated. Negative coping was associated with increased fatigue. SEM revealed that work–family conflict exerted a direct positive effect on occupational fatigue (*β* = 0.637), and both positive (β = −0.360) and negative (*β* = 0.077) coping styles partially mediated this relationship. The model showed an acceptable fit (CFI = 0.853, TLI = 0.837, RMSEA = 0.079).

**Conclusion:**

Occupational fatigue is prevalent among endoscopy nurses in China and is influenced by work–family conflict, coping styles, and social support. Interventions targeting scheduling optimization, the enhancement of social support, and the promotion of adaptive coping strategies are essential to mitigate fatigue and improve workforce well-being.

## 1 Introduction

Occupational fatigue has emerged as a prevalent occupational health issue among nurses globally. It adversely affects not only nurses’ physical and psychological well-being but also impairs their clinical performance, leading to reduced care quality and increased patient safety risks (1, 2). Prior research has linked occupational fatigue to diminished cognitive functioning, lower work efficiency, and a higher likelihood of clinical errors (3).

Occupational fatigue is characterized by both physical and psychological symptoms, such as diminished alertness, sleep disturbances, and emotional exhaustion. While fatigue typically reflects a short-term reaction to workload pressures, it can evolve into chronic burnout if left unaddressed.

With the rapid development of healthcare technologies and the rising demands of patient care, nurses are experiencing increasingly heavy workloads. In China, recent studies report an occupational fatigue rate of 84.96% among nurses (4), significantly exceeding that of physicians and other healthcare professionals, as well as the 75–80% prevalence reported in the United States (5, 6). In Japan, nurses frequently exceed 45 working hours per week, further exacerbating fatigue-related outcomes (7). These international comparisons underscore the critical need for targeted investigations into the specific causes of fatigue among Chinese nurses.

Endoscopy nurses, in particular, are highly susceptible to occupational fatigue due to the demanding nature of their work. Digestive endoscopy procedures require precision, rapid task turnover, prolonged standing, and constant mental focus, all of which contribute to a heightened physical and cognitive workload (8). Despite this, empirical studies on fatigue among endoscopy nurses remain limited, and the mechanisms underlying its development have yet to be fully explored.

Given these challenges, there is a pressing need to investigate both the current status and the contributing factors of occupational fatigue in this population. This study addresses this gap by examining the interplay between work–family conflict, coping styles, and social support in shaping fatigue outcomes among endoscopy nurses in China.

### 1.1 Work characteristics of endoscopy nurses and occupational fatigue

The global demand for gastrointestinal endoscopy has risen significantly in recent years. In China alone, the annual number of procedures has surpassed 14 million and is projected to reach 51 million by 2030 (9). This surge, coupled with an uneven distribution of healthcare resources and a shortage of skilled personnel, has intensified the workload for endoscopy nurses (10).

Compared with general surgical nursing, endoscopy nursing involves more frequent patient turnover, higher procedural volume, and greater technical demands. Nurses are required to maintain prolonged periods of concentration and physical endurance while assisting with complex procedures. The working environment is characterized by high risk and high responsibility, often involving night shifts, emergency care, and irregular hours, all of which contribute to both physical and psychological fatigue (8, 11).

Sustained exposure to high-intensity work can result in musculoskeletal pain, sleep disorders, emotional exhaustion, anxiety, and depression. These symptoms not only compromise nurse well-being but also adversely affect care quality and patient outcomes (8, 12).

### 1.2 Influencing factors of occupational fatigue

Occupational fatigue arises from a complex interaction of individual, organizational, and psychosocial factors. Among these, work–family conflict (WFC) is widely recognized as a significant contributor. According to the Conservation of Resources (COR) theory, when individuals are unable to maintain balance between professional and familial roles, the resulting depletion of resources leads to increased stress and fatigue (13). For endoscopy nurses, the combination of long working hours, irregular shifts, and high-intensity clinical demands often makes it difficult to fulfill family responsibilities, thereby intensifying WFC. Empirical studies have shown that higher levels of work–family conflict are associated with emotional exhaustion, elevated psychological stress, and reduced job satisfaction among nurses (14, 15). In addition, occupational fatigue may further impact family life, creating a recurring cycle of stress that reinforces itself over time (16).

By contrast, social support plays a protective role in mitigating occupational fatigue. Support from family members, colleagues, supervisors, and friends can help reduce work-related stress and enhance psychological resilience (17, 18). Nurses who perceive higher levels of social support tend to report lower levels of fatigue, improved mental health, and greater job satisfaction (19). However, the demanding characteristics of endoscopy nursing, such as frequent night shifts and unpredictable emergencies, often limit opportunities for consistent social interaction and support, thereby increasing the risk of fatigue.

Coping style is another critical factor influencing occupational fatigue. Coping strategies determine how nurses respond to stress and whether they are able to recover lost psychological resources. Positive coping strategies, including seeking assistance, regulating emotions, and managing time effectively, have been associated with reduced psychological burden and lower fatigue levels. In contrast, negative coping responses such as avoidance, denial, and emotional suppression may accelerate the depletion of cognitive and emotional resources, leading to increased fatigue (8, 20). Studies have shown that nurses who adopt adaptive coping strategies are more likely to maintain psychological resilience and experience lower levels of burnout (21, 22).

Based on the COR theory, this study investigated the current status and influencing factors of occupational fatigue among endoscopy nurses in China. A multicenter cross-sectional design was used to explore how work–family conflict, coping strategies, and social support interact to shape fatigue levels. Furthermore, a structural equation model (SEM) was developed to examine whether coping styles mediate the relationship between work–family conflict and occupational fatigue. The findings aim to provide a theoretical foundation and practical guidance for designing effective fatigue-reduction strategies for endoscopy nursing professionals.

## 2 Methods

### 2.1 Study design and participants

This study employed a multicenter cross-sectional design. Data were collected using a standardized electronic questionnaire from July 1 to August 15, 2024. A non-probability sampling strategy was adopted to recruit participants from 26 tertiary hospitals across 14 provincial-level administrative regions in China. All participants were engaged exclusively in gastrointestinal endoscopy-related clinical work and patient care within dedicated endoscopy centers.

Inclusion criteria were as follows:

(1) At least 2 years of continuous experience in endoscopy nursing;
(2) Active clinical service participation for no less than 80 percent of standard working hours during the previous 6 months;
(3) Successful completion of a quality control test, defined as achieving at least 90 percent accuracy on a simulated scale.

Exclusion criteria included:

(1) Recent job transfer or major workflow adjustments within the past 3 months;
(2) Concurrent administrative or teaching duties in nursing management roles;
(3) Comorbidities such as chronic pain syndromes or sleep disorders;
(4) History of psychiatric medication use or psychotherapy.

The study was approved by the Ethics Committee of Deyang People’s Hospital (Approval No. 2023–04-083-K01). It was conducted in accordance with institutional guidelines and local regulations. All participants provided written informed consent before data collection. The study adhered to the ethical standards of the Declaration of Helsinki concerning research involving human subjects. Anonymity and data confidentiality were strictly maintained throughout.

### 2.2 Sample size estimation

The study involved a total of 20 observed variables. According to the conventional sample size estimation principle, the required sample should be 5–10 times the number of variables (23). Considering an anticipated 20 percent invalid or incomplete response rate, the minimum target sample size ranged between 125 and 250 valid participants.

In total, 340 responses were initially collected through Wenjuanxing, a professional online survey platform in China. After a two-step quality control process, eight responses were excluded due to patterns of uniform answering (*n* = 4), missing key variables (*n* = 3), or logical inconsistencies (*n* = 1). Ultimately, 332 valid responses were retained, exceeding the minimum requirement by 32.8 percent.

### 2.3 Data collection procedures

Data were collected using the Wenjuanxing online platform. Initially, the research team established collaborations with head nurses from the participating endoscopy centers. An online briefing was conducted to explain the research objectives, procedures, and data security protocols. Ethical approval and departmental authorization were obtained prior to distribution.

The survey link was disseminated through institutional WeChat groups. To ensure data quality, the following control measures were implemented:

(1) Forced-response settings were applied to avoid missing data;
(2) Duplicate responses were prevented using dual recognition of IP addresses and device IDs;
(3) A minimum and maximum response time threshold (10–30 min) was enforced.

A three-level quality assurance system was applied:

(1) The system automatically screened for irregular response patterns;
(2) Manual checks were performed to verify logical consistency across key variables;
(3) Two independent reviewers conducted cross-validation of suspicious entries.

Questionnaires were excluded if they met any of the following conditions:

(1) More than 20 percent of items were missing;
(2) Inconsistencies in key logic items;
(3) Response time fell outside the preset threshold of 10–60 min.

### 2.4 Measurement instruments

#### 2.4.1 Demographic data

A self-designed questionnaire was used to collect demographic information including gender, age, marital status, education level, professional title, years of service, average daily working hours, number of working days per week, and monthly income.

#### 2.4.2 Occupational fatigue

The Fatigue Assessment Instrument (FAI), developed by Schwartz et al. (24) and translated into Chinese by Wang et al. (25), was used to assess occupational fatigue. The scale comprises 29 items across four dimensions: fatigue severity, situation specificity, fatigue consequences, and responsiveness to rest or sleep. Each item is rated on a seven-point Likert scale (1 = strongly disagree, 7 = strongly agree). Dimension scores are calculated by averaging item responses, with total scores ranging from 4 to 28. Higher scores indicate greater fatigue severity.

The FAI has demonstrated good reliability and validity in prior studies. In the current sample, Cronbach’s alpha for the overall scale was 0.848. Exploratory factor analysis showed strong construct validity, with a KMO value of 0.782 and a significant Bartlett’s test (*χ*^2^ = 4239.102, df = 105, *p* < 0.001), identifying three factors that explained 65.3 percent of the total variance.

#### 2.4.3 Work–family conflict

Work–family conflict was assessed using the Work–Family Conflict Scale developed by Carlson et al. (26) and translated into Chinese by Bai et al. (27). The scale consists of 18 items measuring both work-to-family and family-to-work conflict. Responses are recorded on a five-point Likert scale (1 = strongly disagree, 5 = strongly agree), with total scores ranging from 18 to 90. Higher scores reflect more severe work–family conflict. Cronbach’s alpha in this study was 0.863.

#### 2.4.4 Coping styles

The Simplified Coping Style Questionnaire (SCSQ), originally developed by Folkman and Lazarus (28) and translated by Xie (29), was used to assess individual coping styles. The scale includes 20 items divided into positive coping (12 items) and negative coping (8 items) dimensions. Each item is rated on a four-point scale (0 = never, 3 = frequently). Higher scores reflect more frequent use of the corresponding coping strategy. In this study, Cronbach’s alpha was 0.910 for positive coping.

#### 2.4.5 Social support

Social support was measured using the Perceived Social Support Scale (PSSS) developed by Zimet et al. (30) and translated by Jia and Yue (31). The instrument comprises 12 items across three dimensions: family support, friend support, and support from significant others. A seven-point Likert scale is used (1 = strongly disagree, 7 = strongly agree), with total scores ranging from 12 to 84. Higher scores indicate greater perceived social support. The Cronbach’s alpha in this sample was 0.952.

### 2.5 Statistical analysis

All statistical analyses were performed using STATA version 16.0. Categorical variables were summarized using frequencies and percentages, while continuous variables were presented as means and standard deviations.

To assess potential common method bias, Harman’s single-factor test was conducted. Scale validity was examined using the Kaiser–Meyer–Olkin (KMO) test and Bartlett’s test of sphericity. Univariate analyses (chi-square tests) were performed to assess associations between demographic factors and occupational fatigue. Pearson correlation analysis was used to evaluate relationships among occupational fatigue, work–family conflict, coping styles, and social support.

Hierarchical multiple linear regression was conducted to identify the predictors of occupational fatigue. Variables were entered in sequential blocks, beginning with demographic characteristics, followed by work–family conflict, coping styles, and social support. Structural equation modeling (SEM) with maximum likelihood estimation was used to assess the mediating effects of coping styles. Bootstrap resampling (5,000 samples) and 95 percent bias-corrected confidence intervals were applied to determine the significance of indirect effects. A mediating effect was considered significant if the confidence interval did not include zero. Model fit was evaluated using standard indices including the Comparative Fit Index (CFI), Tucker–Lewis Index (TLI), and Root Mean Square Error of Approximation (RMSEA). Statistical significance was set at *p* < 0.05.

## 3 Results

### 3.1 Testing for common method bias

Harman’s single-factor test was conducted to assess the risk of common method bias. The results showed that the first factor accounted for 15.53 percent of the total variance, which is substantially below the critical threshold of 40 percent. This indicates that common method variance did not pose a serious threat to the validity of the study findings. However, it should be noted that Harman’s single-factor test has limited sensitivity in detecting more subtle method effects. Therefore, the results should be interpreted with caution. To further reduce potential common method bias, several procedural remedies were applied, including anonymous and voluntary participation, the use of validated measurement instruments, and separation of questionnaire items by construct.

### 3.2 Demographic characteristics of participants

A total of 332 valid questionnaires were analyzed. Among the participants, 77 were male (23.19 percent) and 255 were female (76.81 percent). In terms of age distribution, 12.35 percent were under 30 years old, 54.21 percent were between 30 and 39 years old, 23.80 percent were between 40 and 49 years old, and 9.64 percent were aged 50 or older.

Regarding marital status, 86.75 percent were married, 10.84 percent were unmarried, and 2.41 percent were divorced. The majority of participants held a bachelor’s degree (82.23 percent), followed by junior college or below (8.13 percent), and master’s degree or above (9.64 percent). Most held junior professional titles (65.66 percent), while 26.51 percent held intermediate titles and 7.83 percent held senior titles.

In terms of work experience, 36.14 percent had less than 3 years of service, 35.24 percent had 3–5 years, and the remaining were distributed across longer durations. Daily working hours varied, with 10.54 percent working less than 8 h per day and 15.06 percent working more than 12 h. Regarding weekly working days, 37.05 percent worked fewer than 5 days, and 3.01 percent worked 6.5 to 7 days per week. The majority of participants earned between 7,000 and 9,000 yuan per month (49.10 percent).

### 3.3 Occupational fatigue scores of endoscopy nurses

The occupational fatigue scores of 332 endoscopy nurses were 22.075 ± 2.588 points, indicating a relatively high level. Among them, fatigue had the most significant impact on rest and sleep. The scores of each dimension of occupational fatigue are shown in Table 1.

**Table 1:** Occupational fatigue scores of endoscopy nurses (*n* = 332).

Variables	Dimensions/Items	*M*	SD
Occupational fatigue	4	22.075	2.588
Consequences of fatigue	3	5.423	1.278
Situation specificity	6	5.208	0.984
Responsiveness to rest/sleep	2	6.304	0.963
Global fatigue severity	11	5.139	1.044

### 3.4 Univariate analysis of occupational fatigue

The results of the univariate analysis revealed that there were statistically significant differences in occupational fatigue in terms of different age groups, genders, marital statuses, daily working hours, and weekly working days (*p* < 0.05) (refer to Table 2).

**Table 2:** Univariate analysis of occupational fatigue (n=332).

Variables	Items	*N* (%)	Occupational fatigue (*M* ± SD)	*F*/*t*	*P*
Age	<30	41 (12.35%)	21.524 ± 1.490	2.980	0.031
	30–39	180 (54.21%)	21.230 ± 1.827		
	40–49	79 (23.80%)	21.737 ± 2.459		
	≥50	32 (9.64%)	22.267 ± 2.376		
Genders	Male	77 (23.19%)	22.365 ± 2.550	22.210	0.000
	Female	255 (76.81%)	21.153 ± 1.772		
Marital statuses	Not married	36 (10.84%)	21.369 ± 2.246	3.390	0.035
	Married	288 (86.75%)	21.425 ± 2.024		
	Divorced	8 (2.41%)	20.528 ± 1.697		
Daily working hours	8 h	35 (10.54%)	21.604 ± 1.420	4.140	0.003
	8–9 h	81 (24.40%)	21.267 ± 1.970		
	9–10 h	76 (22.89%)	21.240 ± 2.585		
	10–12 h	90 (27.11%)	22.366 ± 2.346		
	>12 h	50 (15.06%)	22.151 ± 2.215		
Weekly working days	<5d	123 (37.05%)	20.938 ± 1.928	7.440	0.000
	5–5.5d	122 (36.74%)	21.167 ± 1.573		
	5.5–6d	55 (16.57%)	22.344 ± 2.514		
	6–6.5d	22 (6.63%)	22.365 ± 2.078		
	6.5–7d	10 (3.01%)	22.520 ± 2.958		

### 3.5 Correlation analysis of work–family conflict, coping styles, social support, and occupational fatigue

The results of the Pearson correlation analysis showed that work-family conflict (*r* = 0.192, *p* < 0.05) and negative coping styles (*r* = 0.233, *p* < 0.05) were positively correlated with occupational fatigue, which could exacerbate occupational fatigue. On the contrary, positive coping styles (*r* = −0.357, *p* < 0.05) and social support (*r* = −0.303, *p* < 0.05) were negatively correlated with occupational fatigue, which could reduce the level of occupational fatigue (see Table 3).

**Table 3:** Scores of work–family conflict, coping styles, and social support among endoscopy nurses and their correlation analysis with occupational fatigue (*n* = 332).

Variables	*M*	SD	Association with occupational fatigue
Work–family conflict	55.087	10.25	0.192*
Positive coping styles	22.461	6.851	−0.357*
Negative coping styles	12.952	5.117	0.233*
Social support	57.473	17.374	−0.303*

*p < 0.05.

### 3.6 Hierarchical regression analysis

A hierarchical regression analysis was conducted with the total score of occupational fatigue as the dependent variable and the variables with statistical significance in the univariate and correlation analyses as the independent variables. The variable coding is presented in Table 4.

**Table 4:** Variable coding of independent variables.

Variables	Variable coding
Age	Actual age
Gender	Male = 1; Female = 2
Marital statuses	Not married = 1; Married = 2; Divorced = 3
Daily working hours	<8 h = 1; 8–9 h = 2; 9–10 h = 3; 10–12 h = 4; >12 h = 5
Weekly working days	<5d = 1; 5–5.5d = 2; 5.5–6d = 3; 6–6.5d = 4; 6.5–7d = 5

In the first layer, general demographic data were included. The variables were gender, age, marital statuses, daily working hours, and weekly working days, which could explain 33.3% of the total variance in occupational fatigue.

In the second layer, work-family conflict was added, and the cumulative explained variance of the total variance reached 41.6%.

In the third layer, positive and negative coping styles were incorporated, and the cumulative explained variance of the total variance was 50.3%. The results are shown in Table 5.

**Table 5:** Hierarchical regression analysis of occupational fatigue among endoscopy nurses (*n* = 332).

Variables	Model 1				Model 2				Model 3			
	*β*	SE	*T*	*P*	*β*	SE	*T*	*P*	*β*	SE	*T*	*P*
Constant	14.458	1.130	12.790	0.000	10.244	1.227	8.350	0.000	13.608	1.357	10.030	0.000
Age	0.119	0.023	5.250	0.000	0.109	0.021	5.130	0.000	0.115	0.020	5.800	0.000
Daily working hours	0.696	0.157	4.450	0.000	0.675	0.147	4.600	0.000	0.687	0.137	5.020	0.000
Weekly working days	0.906	0.173	5.220	0.000	0.834	0.163	5.120	0.000	0.625	0.154	4.070	0.000
Marital statuses	−0.209	0.333	−0.630	0.531	−0.130	0.312	−0.420	0.676	−0.072	0.291	−0.250	0.804
Gender	−0.285	0.277	−1.030	0.305	−0.158	0.261	−0.610	0.544	−0.084	0.242	−0.350	0.729
Work–family conflict					0.083	0.012	6.800	0.000	0.059	0.012	5.070	0.000
Positive coping styles									−0.079	0.016	−4.930	0.000
Negative coping styles									0.054	0.021	2.620	0.009
Social support									−0.019	0.006	−3.110	0.002
*F*	32.530*				38.580*				36.270*			
*R^2^*	0.333				0.416				0.503			

*P < 0.05.

### 3.7 Structural equation modeling

**Figure 1:**
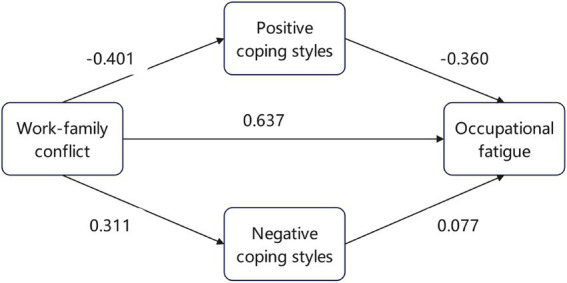
The diagram of the mediating effect model.

This study hypothesized that different coping styles play a mediating role between nurses’ work-family conflict and occupational fatigue. The work-family conflict was regarded as the independent variable, positive coping and negative coping as parallel mediating variables, and occupational fatigue as the dependent variable, all of which were incorporated into the pre-set model. Table 6 presents the model fit indices, which indicate that the structural model achieved an acceptable fit to the data. The diagram of the mediating effect model is shown in Figure 1. The Bootstrap method was used to test the mediating effect. The results are presented in Table 7. The total effect, direct effect, and indirect effect of work-family conflict on occupational fatigue were significant (*p* < 0.05). That is, coping styles play a partial mediating role between work-family conflict and occupational fatigue (*p* < 0.05).

**Table 6:** Summary of structural model fit indices.

Fit index	Value
*χ* ^2^	410.25
df	150
*χ*^2^/df	2.73
CFI	0.853
TLI	0.837
RMSEA	0.079

**Table 7:** Mediating effect test using the bootstrap method (standardized).

Variables	Items	Size of effect	Relative effect value	95%CI	*P*
Positive coping styles	Direct effect	0.007	12.07	0.002–0.013	0.010
	Indirect effect	0.051	87.93	0.015–0.087	0.006
	Total effect	0.058	100	0.022–0.095	0.002
Negative coping styles	Direct effect	0.020	34.48	0.002–0.039	0.027
	Indirect effect	0.038	65.51	0.002–0.074	0.039
	Total effect	0.058	100	0.021–0.095	0.002

## 4 Discussion

This study, grounded in the Conservation of Resources (COR) theory, conceptualizes occupational fatigue as a manifestation of sustained resource depletion in the absence of adequate replenishment. Stressors such as work–family conflict, insufficient social support, and maladaptive coping strategies contribute to this imbalance and ultimately lead to cumulative fatigue. The following sections discuss the key findings in relation to existing literature and theoretical frameworks.

### 4.1 Current status of occupational fatigue among endoscopy nurses

This study shows that endoscopy nurses have a relatively high total score of occupational fatigue, with a particularly prominent score in the dimension of fatigue’s reaction to rest/sleep, which is higher than the levels reported by Zhang et al. (32) among surgical nurses (19.83 ± 3.67) and Zeng et al. (8) among endoscopy nurses in parts of China (17.73 ± 5.64). Based on the Conservation of Resources Theory, the reasons are analyzed as follows: Excessive resource depletion caused by workload: High-intensity endoscopy diagnosis and treatment require nurses to maintain high concentration throughout the process. Their physical and mental states are constantly in a tense condition, leading to excessive consumption of energy and psychological resources (33). The shift-work system, especially night shifts, disrupts the biological clock and damages sleep resources. Long-term sleep deprivation causes fatigue to accumulate, resulting in abnormal rest/sleep reactions. Accelerated resource loss due to psychological stress: Endoscopy operations involve high risks. Nurses are worried about being held accountable for mistakes, and long-term anxiety consumes a large amount of psychological resources. When communicating with patients, they may face patients’ fear and accusations, and negative emotions may further deplete psychological resources, potentially increasing fatigue and impairing recovery through rest (34). Obstacles to resource acquisition due to hampered career development: endoscopy nurses have limited promotion opportunities and narrow promotion channels, making it difficult for them to gain a sense of achievement and resources for growth. The lack of career planning may be linked to lower work motivation and resource loss, contributing to higher levels of job burnout and fatigue (35). Weakened resource reserves due to insufficient social support: At home, the special nature of their work makes it difficult for nurses to balance family life, leading to more family conflicts and a decrease in emotional support resources. In society, the public has insufficient awareness of the importance of their work and low recognition, resulting in a shortage of respect and recognition resources. Weakened resource reserves may reduce stress-buffering capacity, potentially contributing to a cycle of persistent fatigue (36).

In view of this, managers need to adopt a multi-pronged approach. Comprehensive measures should be taken in terms of reasonable work arrangement, reduction of psychological burden, expansion of career paths, optimization of the work environment, and enhancement of social support to improve the current situation of occupational fatigue among endoscopy nurses.

### 4.2 Work–family conflict and the mediating role of coping styles

#### 4.2.1 Work–family conflict as a risk factor

Consistent with COR theory, the study confirmed that work–family conflict significantly contributes to occupational fatigue. Endoscopy nurses are frequently required to alternate between demanding professional roles and familial responsibilities. This continuous role switching drains time, energy, and emotional resources (37).

The emotional impact of role conflict includes anxiety, guilt, and frustration. Nurses may feel tension from being unable to fulfill family obligations due to the demands of high-risk procedures. These negative emotions may be associated with reduced psychological resilience and increased fatigue (38).

Furthermore, sustained work–family conflict limits opportunities for resource acquisition. Time constraints prevent engagement in professional development or social interactions, thereby narrowing access to external resources such as support, training, or peer recognition. As the conflict persists, fatigue levels increase accordingly (39, 40).

To mitigate this effect, management strategies should include flexible scheduling systems, such as self-scheduling or shift preference mechanisms. Additionally, family-friendly policies, childcare support, and counseling resources should be considered to reduce dual-role strain.

#### 4.2.2 Mediating role of coping styles

The study found that both positive and negative coping styles partially mediated the relationship between work–family conflict and occupational fatigue. This finding aligns with COR theory, which emphasizes the role of coping behavior in the management of stress and resource loss.

Positive coping strategies, such as emotional regulation, problem-solving, and seeking support, enable individuals to buffer resource depletion and regain control over stressful situations (8, 41). Nurses who actively employ these strategies are more likely to recover psychological resources and resist the progression of fatigue.

However, positive coping also requires a baseline level of emotional resilience and access to support, which may not be available to all individuals. This could limit the strength of its mediating effect.

In contrast, negative coping strategies, such as avoidance or emotional suppression, tend to prolong or exacerbate stress. These maladaptive behaviors may hinder resource recovery and be linked to persistent fatigue (42). Negative emotions can also damage interpersonal relationships and reduce perceived social support, further impairing the capacity to recover.

From a managerial perspective, structured psychological support systems, such as peer mentoring, stress management workshops, and confidential counseling, can help nurses build and sustain positive coping skills.

### 4.3 Influence of demographic characteristics

The results of this study show that the gender, age, marital status, daily working hours, and weekly working days of endoscopy nurses are statistically significant in all three models of the regression analysis.

#### 4.3.1 Gender differences

According to the Conservation of Resources theory, social and cultural expectations may place greater pressure on men to assume the role of primary economic providers within families (43). As a result, male endoscopy nurses may invest more time and energy in their professional roles, which could be associated with higher levels of resource depletion. In addition, traditional gender norms may influence coping behaviors, with men being less likely to seek external emotional or social support when facing work-related stress (44, 45), potentially limiting their opportunities for resource replenishment.

However, these interpretations should be viewed with caution, as gender differences in occupational fatigue may also be influenced by unmeasured factors such as task allocation, workload intensity, shift patterns, and organizational roles. Therefore, the observed gender differences in fatigue levels likely reflect a complex interaction of social, occupational, and individual factors rather than gender-related characteristics alone.

#### 4.3.2 Age

As age increases, physical functions decline, and the reserve of physiological resources decreases, reducing the ability to cope with high-intensity work (46). At the same time, senior nurses with rich experience take on more complex tasks and responsibilities and need to invest more cognitive and emotional resources. Long-term work stress can easily lead to job burnout, resulting in a shortage of psychological resources. Thus, there is a positive correlation between age and occupational fatigue.

#### 4.3.3 Marital statuses

Married nurses need to balance more affairs between work and family. While facing work tasks, they also have to handle family chores and take care of family members, which leads to the dispersion and intensified consumption of resources (47). Unmarried nurses have lighter family burdens and can concentrate more resources on work. Divorced nurses have relatively simple family relationships and relatively less resource depletion. Therefore, married nurses have a higher level of occupational fatigue.

#### 4.3.4 Working hours

An increase in daily working hours and weekly working days means that nurses are continuously exposed to work-related stressors, and their physical and mental resources are constantly being consumed (48). Extended working hours may reduce time for rest and recovery, potentially leading to sustained resource depletion and elevated fatigue levels (49).

Based on the above findings, managers should pay attention to gender differences, guide men to seek help actively, and broaden resource replenishment channels; attach importance to age stratification, adjust job positions reasonably, and establish a health-care mechanism; arrange work schedules scientifically, optimize work processes to improve efficiency and reduce the workload; and encourage nurses to take breaks and relax during work to promote resource recovery.

### 4.4 Role of social support

Social support was negatively correlated with occupational fatigue, reinforcing its role as a protective factor. According to COR theory, emotional and material support can buffer resource loss and facilitate recovery from stress (50).

Support from family members can reduce psychological burden and provide emotional stability (51, 52). Shared responsibilities at home help conserve energy for professional duties. Friends and colleagues provide informational and emotional support, promote social belonging, and facilitate psychological resilience (53).

Support from mentors or senior professionals contributes to skill development and enhances professional identity, helping nurses build internal resources and confidence (54).

Healthcare administrators should recognize the protective role of social support and take proactive steps to establish structured support mechanisms, such as team-based reflection sessions, professional development mentoring, and formal recognition programs.

## 5 Conclusion

This study revealed a high prevalence of occupational fatigue among endoscopy nurses in China, particularly in the domain of rest and sleep responsiveness. Drawing upon the Conservation of Resources (COR) theory, the findings demonstrate that work–family conflict, coping styles, and social support significantly influence the level of occupational fatigue in this population.

Work–family conflict was positively associated with fatigue and may also be linked to increased fatigue severity via maladaptive coping strategies. In contrast, positive coping strategies and higher levels of social support served as protective factors, reducing the risk and intensity of fatigue.

These results underscore the need for targeted interventions aimed at reducing occupational fatigue among endoscopy nurses. Practical strategies may include flexible scheduling systems, structured coping skills training, mental health support programs, and the development of supportive workplace environments. Hospital administrators and policymakers should prioritize fatigue management in order to protect nurse well-being, maintain care quality, and ensure patient safety.

Furthermore, this study expands the application of COR theory in clinical nursing by elucidating the mediating role of coping styles in the relationship between work–family conflict and fatigue. The proposed structural equation model offers a theoretical foundation for future empirical research and intervention development in occupational health.

## 6 Limitations

This study has several limitations.

First, due to the cross-sectional design, temporal sequencing of variables cannot be confirmed. Therefore, interpretations of directionality or causality should be made with caution. In addition, although the structural equation model demonstrated acceptable fit, the fit indices (e.g., CFI and TLI) did not reach the conventional thresholds for excellent model fit. This should be considered when interpreting the strength and stability of the structural relationships. Longitudinal research is needed to better assess causal pathways and validate the proposed model.

Second, although procedural remedies were applied to mitigate common method bias, the use of Harman’s test alone may not fully capture all potential method effects, which remains a methodological limitation.

Third, the study employed a non-probability sampling strategy, and all participants were recruited from tertiary hospitals through online platforms. This may introduce selection bias and limit the generalizability of the findings. Therefore, caution should be exercised in extending the results to nurses working in secondary hospitals, rural settings, or non-endoscopy departments.

## Data Availability

The raw data supporting the conclusions of this article will be made available by the authors, without undue reservation.
